# Governance ‘tool kits’ for universal health coverage in India: guidelines for implementing the Expert Group’s recommendations

**DOI:** 10.1186/1753-6561-6-S5-O8

**Published:** 2012-09-28

**Authors:** VR Raman, Kabir Sheikh, Prasanna Saligram, Namrata Verma, Nikhil Sharma

**Affiliations:** 1 Health Governance Hub, Public Health Foundation of India, Delhi, India; 2 Centre of Public Health, Panjab University, Chandigarh, India

## Introduction

Serious efforts are taking place worldwide to attain universal health coverage (UHC). In India, although an unattained goal for long, recent recommendations of the High Level Expert Group (HLEG) instituted by the Planning Commission have brought the UHC agenda to its zenith [1]. However, actualisation of HLEG recommendations requires an in-depth understanding of the structural and operational reforms to the health system implied in each recommendation, and the outlining of a detailed implementation framework. Given the widespread limitations of health planning capacities, it was felt important to provide states with ‘toolkits’ to realise the UHC recommendations.

## Methods

The toolkits were developed by a multidisciplinary team of experts specialised in planning, implementation and evaluation of health systems. Each of the recommendations was elaborated into a set of action points, signifying the specific institutional reforms and measures required for their realization. In doing so, lessons were also drawn from comparable national and international experiences. The action points were verified by experts and implementers and developed in the form of a tabular framework, with sub-activities against each action point, a list of stakeholders and a timeframe. While articulating all necessary reforms clearly and specifically in the toolkit, we also accorded care to preserve the flexibility required at different implementation levels. This was to avoid being overtly prescriptive, while at the same time providing focused guidance for application in varying settings. It was also found to be important to provide guidance on the ‘how’ of the implementation, in addition to the ‘what’.

## Results

The project is at an interim stage wherein we have finalised the toolkit for the recommendations on community participation and citizen engagement, out of all the recommendations of the HLEG. The work on other recommendations is progressing. We observe that a systematic analysis of each recommendation opens up a large horizon of implementation challenges. Each recommendation leads into several action points and numerous sub-activities at both national and state level. These include, but are not restricted to devising rules and norms, establishing institutions, employing personnel, undertaking trainings and securing and deploying finances. It is estimated that it will take 18-24 months at the national level, and 48-60 months at the state level, to institute preparatory reforms that are requisite for implementing the UHC recommendations. This may be exceeded in instances where capacities at state level are less developed, or for larger-than-average states. There is a simultaneous need to set into motion local and community-led processes, which in turn necessitate massive capacity-building efforts. The toolkit reflects a paradigm shift from the current trend of centralised technical assistance and out-sourcing, towards self-reliance.

## Discussion

In the case of most nationally mandated programmes and reforms, it is widely observed that the lack of adequate detail in implementation guidelines or, conversely, overly top-down or prescriptive guidelines create varied challenges and limitations for the implementers. Crucially, the important step of dismantling or modifying existing institutions is rarely addressed, when institutional or structural reforms are propagated. Newer systems are often layered onto existing decision-making structures creating parallel implementation chains. Further, the establishment of systems of governance is seldom prioritised while instituting reforms, nor is the ethical basis of such governance typically articulated. All these deficits in the reform process result in poor health governance and eventually reflect in health outcomes. In the context of UHC, it is important to have a robust framework that guides governance in an integrated manner, and facilitates better implementation at central and state level. The governance toolkit is one such robust, sensitive and practical approach.

The limitations of the toolkit in its present iteration include that it is restricted to the national and state/UT levels only - yet it contains the potential to be expanded to district, block and peripheral levels. The toolkit has potential to support all levels of decision makers to adopt measures and to ensure better implementation of UHC at their respective levels, and to ascertain the efficacy of these measures.

## Competing interests

Authors declare that they have no conflict of interest.

## Funding statement

The study was funded by Oxfam India, New Delhi for the Initial phase of “Preparing the Governance Toolkit for Universal Health Coverage” project.
